# Insights into Native Single-Atom Electrocatalyst Site Structures

**DOI:** 10.1021/acsnano.6c03527

**Published:** 2026-06-06

**Authors:** Michael J. Zachman, Hasnain Hafiz, Colum M. O’Leary, Chaewon Lim, Dong Young Chung, Sirui Li, Subin Park, Jiheon Kim, David A. Cullen, Edward F. Holby, Vojislav R. Stamenkovic

**Affiliations:** † Theoretical Division, Los Alamos National Laboratory, Los Alamos, New Mexico 87545, United States; ‡ Department of Physics and Astronomy and California Nano Systems Institute, University of California Los Angeles, Los Angeles, California 90095, United States; § Department of Chemical and Biomolecular Engineering, University of California, Irvine, California 92697, United States; ∥ Materials Science Division, Argonne National Laboratory, Argonne, Illinois 60439, United States; ⊥ Department of Chemical and Biomolecular Engineering, Korea Advanced Institute of Science and Technology (KAIST), Daejeon 34141, Republic of Korea; # School of Chemical and Biological Engineering, Seoul National University (SNU), Seoul 08826, Republic of Korea; ∇ Center for Nanophase Materials Sciences, Oak Ridge National Laboratory, Oak Ridge 37831, Tennessee, United States; ○ Department of Chemistry, University of California, Irvine 92697, California, United States

**Keywords:** single-atom electrocatalysts, atomic-scale structure, high-resolution imaging, 4D-STEM, ultralow voltage, electron ptychography

## Abstract

Single-atom electrocatalysts consisting of metal atoms embedded in a carbon matrix are promising next-generation catalysts for green hydrogen production and utilization, CO_2_ reduction, low-temperature CO oxidation, ammonia production, plastic decomposition, and electrochemical energy storage. The origins of activity and stability for the single-atom sites are still debatable, however, because of constrained insights into their local structure resulting from idealized models and experiments derived from a large number of individual sites. Insights into structural variations around single atomic sites are therefore critical for the continued development of these next-generation catalysts. While electron microscopy commonly provides atomic-scale information about these materials, the beam sensitivity of individual sites makes structural determination by conventional low-voltage (60 keV) techniques challenging. Here, we introduce ultralow-voltage electron ptychography, performed at 30 keV, that enables determination of the lattice structure around individual metal sites in a well-defined single-atom electrocatalyst system while essentially eliminating knock-on structural modifications. Pairing these atomic-scale, site-specific measurements with computational methods will broaden our understanding of the activity and stability of these materials, which will accelerate the development of the next generation of catalysts.

## Graphical abstract

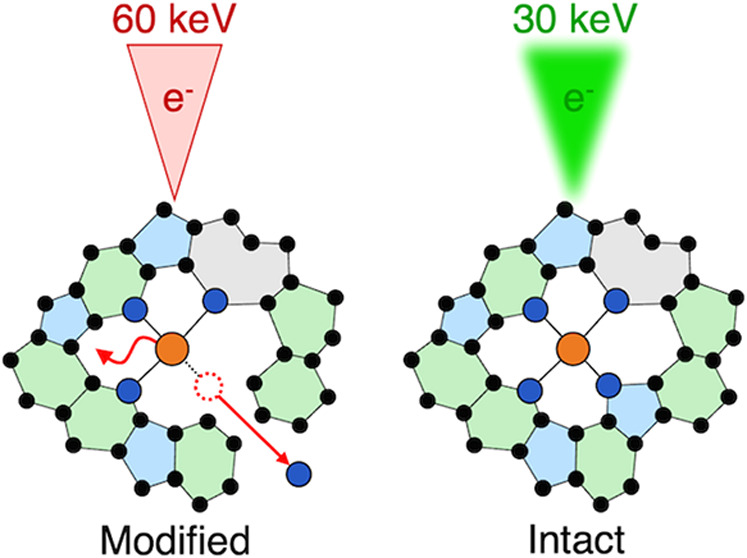

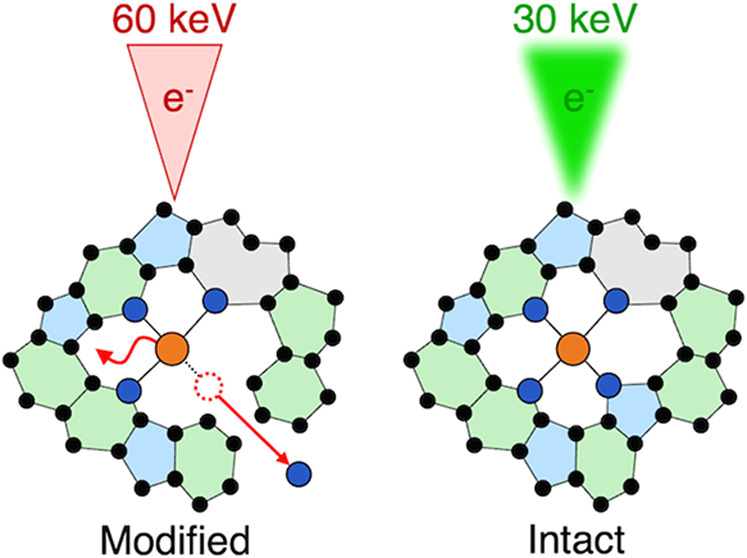

## Introduction

Single metal atoms embedded in a carbon matrix have the potential to transform various technologies necessary for achieving a sustainable future, such as electrochemical energy storage and conversion,^1–4^ waste decomposition,^5^ CO_2_ abatement,^6^ and ammonia production.^7^ Bulk measurements of average active site properties in these carbon-hosted metal single-site (CMS) catalysts, also referred to as atomically dispersed,^3^ single-site,^8^ or single-atom^6,7^ catalysts, can be provided by a variety of methods such as X-ray photoelectron spectroscopy,^9^ X-ray absorption spectroscopy,^10^ and Mössbauer spectroscopy,^11^ While these measurements provide valuable information about averaged, idealized atomic-scale active site structures, they also suggest that local site-to-site variations in structure are present that cannot be directly observed using these techniques. Understanding these local, atomic-scale lattice variations around individual sites and the effect they have on catalyst performance and stability is critical for developing next-generation materials.^10–20^

High-resolution scanning transmission electron microscopy (STEM) and electron energy-loss spectroscopy (EELS) have proven valuable for establishing the dispersion and coordination of metal atoms in CMS materials due to the atomic-scale nature of these sites.^21–26^ Conventional STEM techniques have limited ability to directly image the heavier metal atom and lighter surrounding lattice structure simultaneously, however, due to contrast mechanisms that either depend strongly on atomic number, as with high-angle annular dark-field (HAADF) STEM,^27^ or produce little signal for very thin specimens along with contrast that is difficult to interpret, as with bright-field (BF)-based STEM techniques.^28,29^ Moreover, typical “low-voltage” beam parameters (*e.g*., 60 keV) commonly used to avoid beam-induced damage in two-dimensional (2D) materials readily alter CMS active site structures, as these sites are essentially atomic-scale lattice defects, which are highly beam-sensitve.^30,31^ As a result, not only is imaging of the full CMS site structure by conventional electron microscopy challenging, but reliable determination of the native atomic-scale structure of these sites is not possible using conventional low-voltage beam conditions. To provide an accurate picture of individual CMS site structures, a method that simultaneously produces interpretable contrast over a wide range of atomic numbers while minimizing structural modifications is therefore required. Pairing experimental observations of site structures from such a technique with computational methods like density functional theory (DFT) and molecular dynamics (MD) would provide a new understanding of the relationship between the range of individual site structures present in a CMS catalyst and the performance and stability of the material, accelerating development of next-generation catalysts for a host of applications.

Here, we report a 30 keV electron ptychography technique that allows the native, undisturbed atomic-scale structure of CMS sites to be directly observed, revealing light and heavy elements simultaneously, building on pioneering demonstrations of the feasibility of these ultralow-voltage techniques for visualization of atomic-scale features in systems consisting of heavier atoms.^32,33^ We employ this technique to investigate a well-defined CMS system, a platinum group metal (PGM)-free catalyst containing single-atom Fe sites within a graphitic lattice (Fe–N–C) with exposed monolayer graphene regions. Such CMS materials,^3,20,34–38^ with single Fe atom sites, are a promising class of catalyst for the oxygen reduction reaction (ORR), achieving, in certain cases, activity comparable to that of state-of-the-art Pt-based catalysts. As we demonstrate, ultralow-voltage electron ptychography performed at 30 keV on these single Fe atom sites embedded in graphene provides a unique opportunity to gain insights into the origin of structure–function relationships on a site-by-site basis through direct probing of individual site structures.

## Results and Discussion

Figure 1a shows a schematic of the experimental setup used to obtain conventional annular dark-field (ADF) and four-dimensional (4D) STEM data, the latter of which enables techniques such as differential phase contrast^39^ (DPC) and center-of-mass^40,41^ (CoM) imaging to be performed. These 4D-STEM techniques have recently attracted significant attention due to the approximately linear dependence of their contrast on atomic number for thin samples, which is more interpretable than that of BF-based STEM techniques and provides a meaningful signal for both light and heavy elements, unlike ADF-STEM. Figure 1b,c, for example, compares ADF- and CoM-STEM images generated by multislice electron scattering calculations under typical low-accelerating-voltage conditions (60 keV) for an idealized proposed CMS active site structure anticipated to be present in our model Fe–N–C system.^3,10–12,18,20^ While the lattice structure around the metal site is difficult to detect by conventional ADF imaging due to its insensitivity to light elements, the metal atom itself has high intensity, making ADF an effective tool for rapidly locating the position of metal sites. CoM-STEM, on the other hand, displays both the heavy metal atom and surrounding light-element lattice simultaneously, visualized here through the divergence of the CoM measurements (dCoM), which represents the projected charge density of a single atomic-layer-thin sample convolved with the STEM probe function profile. While a commonly utilized alternative visualization method, integrated CoM (iCoM), maps the projected potential of thin samples similarly to ptychography, we found that dCoM images more directly provide the high-spatial frequency, atomic-scale contrast required here (see Figure S1). The structural information obtained by these techniques can then be additionally supplemented by EELS to confirm the local presence of certain elements, as shown in Figure S2.

**Figure 1:**
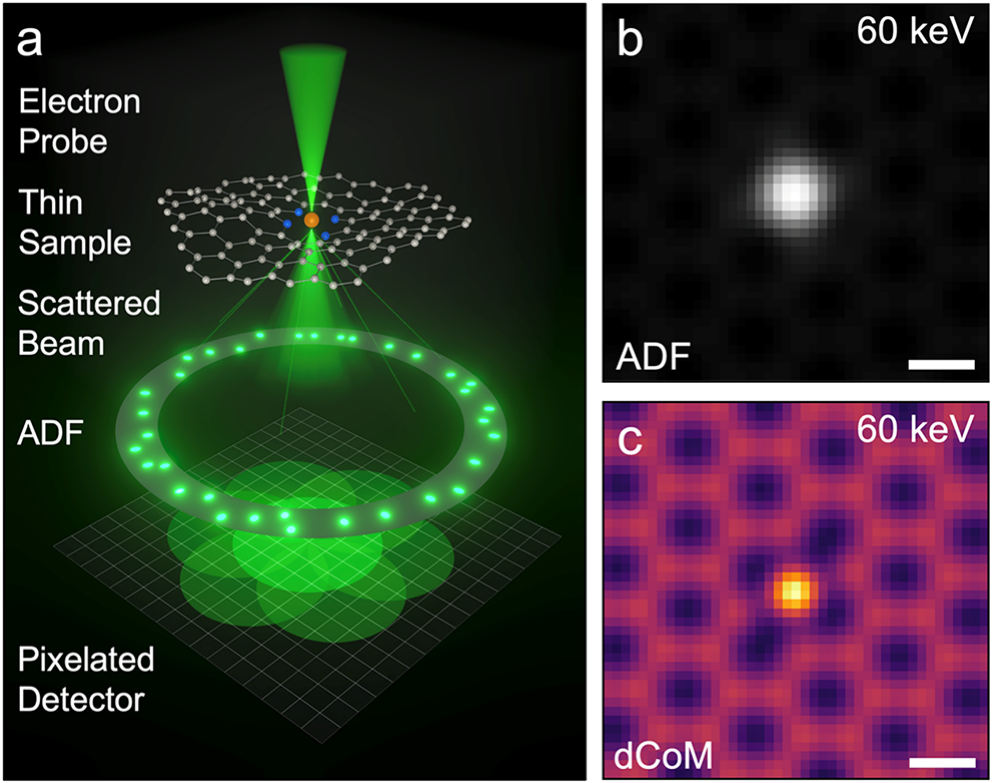
Schematic of experimental setup and simulated 60 keV ADF- and dCoM-STEM images for an idealized proposed CMS active site structure. (a) At each probe position, the STEM probe is focused on the thin sample, individual electrons scattered to high angles are detected and summed on a conventional monolithic ADF detector, and a diffraction pattern is recorded in 2D on a pixelated detector. (b–c) 60 keV multislice electron scattering calculations for an idealized proposed CMS active site structure composed of a single metal atom (Fe here) embedded in a graphitic network show that ADF is significantly less sensitive to light elements than the 4D-STEM techniques, which pixelated detectors enable, such as dCoM. Note that a ∼0.5 Å Gaussian filter was applied to qualitatively approximate the appearance of experimental data, which is blurred, for example, by source-size broadening and residual aberrations. Scale bars, 2 Å.

Given their advantageous contrast mechanisms, 4D-STEM imaging techniques have the potential to experimentally reveal the nature of unique individual CMS site structures. To explore this capability, we performed dCoM imaging on our well-defined catalyst based on Fe atoms embedded in graphene sheets. This material was synthesized to facilitate imaging while remaining an ORR active catalyst, as demonstrated in the polarization curves of Figure S3, and containing representative site structures. Furthermore, samples with and without Fe were produced, Fe–N–C and N–C, respectively, and their polarization curves unequivocally demonstrated that Fe is crucial for high activity under both acidic and alkaline conditions. dCoM imaging confirmed that this Fe is present as individual atoms incorporated into the graphitic lattice, as shown in Figure 2a–d. A variety of site structures were revealed, however, some of which varied significantly from proposed idealized structures. Across all of our experiments, repeated individual site structures were almost never observed. Though the scope of our observations was limited, this suggests the vast breadth of site structures that are likely present in these materials. Additional examples of structure observed are shown in Figure S4. Since the activity, selectivity, and durability of individual CMS sites are highly sensitive to the local coordination environment, especially the structure of carbon rings adjacent to the central metal atom, and the corresponding electronic structure of the sites,^10–20^ ensuring that these structures are native to the material and not artifacts of the imaging process is critical for further optimization of CMS materials’ catalytic properties.

**Figure 2:**
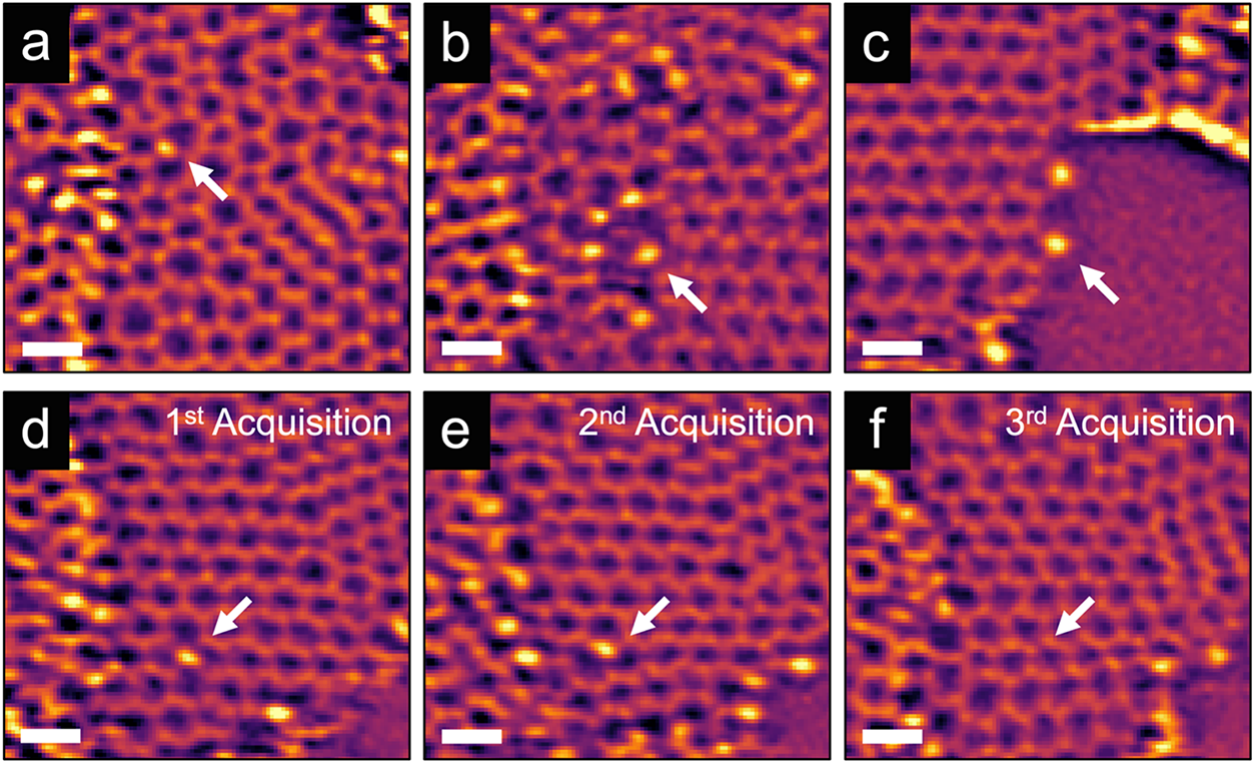
Examples of site structures in a well-defined CMS catalyst based on Fe embedded in a graphene layer and structural modification by 60 keV dCoM imaging. A variety of site structures are observed in CMS catalysts, such as basal plane-embedded (a) 3-fold and (b) more complex structures, as well as (c) edge-supported structures. The beneficial contrast mechanism of these techniques also allows a second graphitic layer, commensurate with the single layer, to be identified on the left, as well as additional Fe atoms in this layer. (d–f) Even for energetically favorable sites, interactions with a 60 keV probe modify structures rapidly, complicating definitive structural determination. Scale bars, 5 Å.

While CoM-STEM allows the light and heavy elements of CMS sites to be imaged simultaneously, we found that the conventional low-voltage 60 keV probe could readily modify the sample structure around the sensitive metal sites, as shown, for example, in the series of acquisitions on a single site shown in Figure 2d–f. These structural modifications rapidly change the local carbon structure around the sites and ultimately result in the removal of the metal atom with occasional replacement by lighter atoms. As a result, structures determined using conventional low-voltage operating conditions may not be accurate, and minimizing these structural modifications is therefore critical for understanding the native active-site structures in CMS materials.

To explore how to minimize sample modifications while imaging, we used a combined *ab initio* molecular dynamics–density functional tight-binding molecular dynamics (AIMD-DFTBMD) approach for calculating electron beam energy thresholds (EBET) for knock-on displacement of atoms from specific site structures (see 4Methods for details).^31^ For electron conductive materials such as graphitic carbon, time scales for relaxation of excitations, for example of core-level electrons, are short enough that modifications to sample structure by the electron probe are mainly due to transfer of kinetic energy from the incident electrons to the sample, which can result in so-called “knock-on damage.” ^42,43^ To directly image the inherent structure of conductive materials without structural modifications, the probe energy must therefore be reduced such that kinetic energy sufficient to break bonds cannot be transferred to the sample. Below this threshold beam energy, modifications to the sample will be nearly entirely suppressed, essentially eliminating any dependence of knock-on damage on dose rate, probe size, imaging technique, or total acquisition time for imaging. Our AIMD-DFTBMD approach allows us to establish which probe energies should allow this knock-on modification-free imaging of native CMS site structures. This method takes the effects of thermal atomic motions into account, which broaden the well-defined threshold energies of static lattices to a distribution of threshold energies that depend on the specific atomic configuration at the moment of electron impact, giving the EBET a temperature dependence^30^ (Figure 3a, Figure S5). Distributions of threshold probe energies for various structures relevant to CMS catalysts were generated using these AIMD-DFTBMD calculations. In general, nitrogen atoms in graphitic (or graphene) structures have the lowest knock-on displacement energy,^30,44,45^ and our results here thus represent knock-on damage of these atoms. By utilizing the calculated EBET distributions, reasonable estimates for the overall probability of a knock-on event occurring within a given data set acquisition can be estimated for various site structures. To do this, the probability that a given incident electron at a certain energy does not result in a knock-on event is calculated from the EBET distribution. A cumulative probability that no knock-on event occurs at a given energy during a full acquisition is then generated by raising the individual electron probabilities to the power of the total number of relevant incident electrons in the acquisition (independent events), calculated using relevant instrument and sample parameters, such as probe current, pixel size, dwell time, and the area over which the probe can interact with susceptible atoms. The cumulative probability that a knock-on event occurs during an acquisition at a given probe energy for a given set of experimental parameters can then be directly calculated by subtracting this distribution from unity. For additional details, see the 4Methods section. Overall knock-on probability distributions for two relevant model site structures calculated by this method are shown in Figure 3b. The results for a basal plane-hosted FeN_4_C_10_ site show that a 60 keV electron probe has greater than a 50% chance of displacing a nitrogen atom in any given acquisition, which is consistent with our observations, as shown in Figure 2d–f.

**Figure 3:**
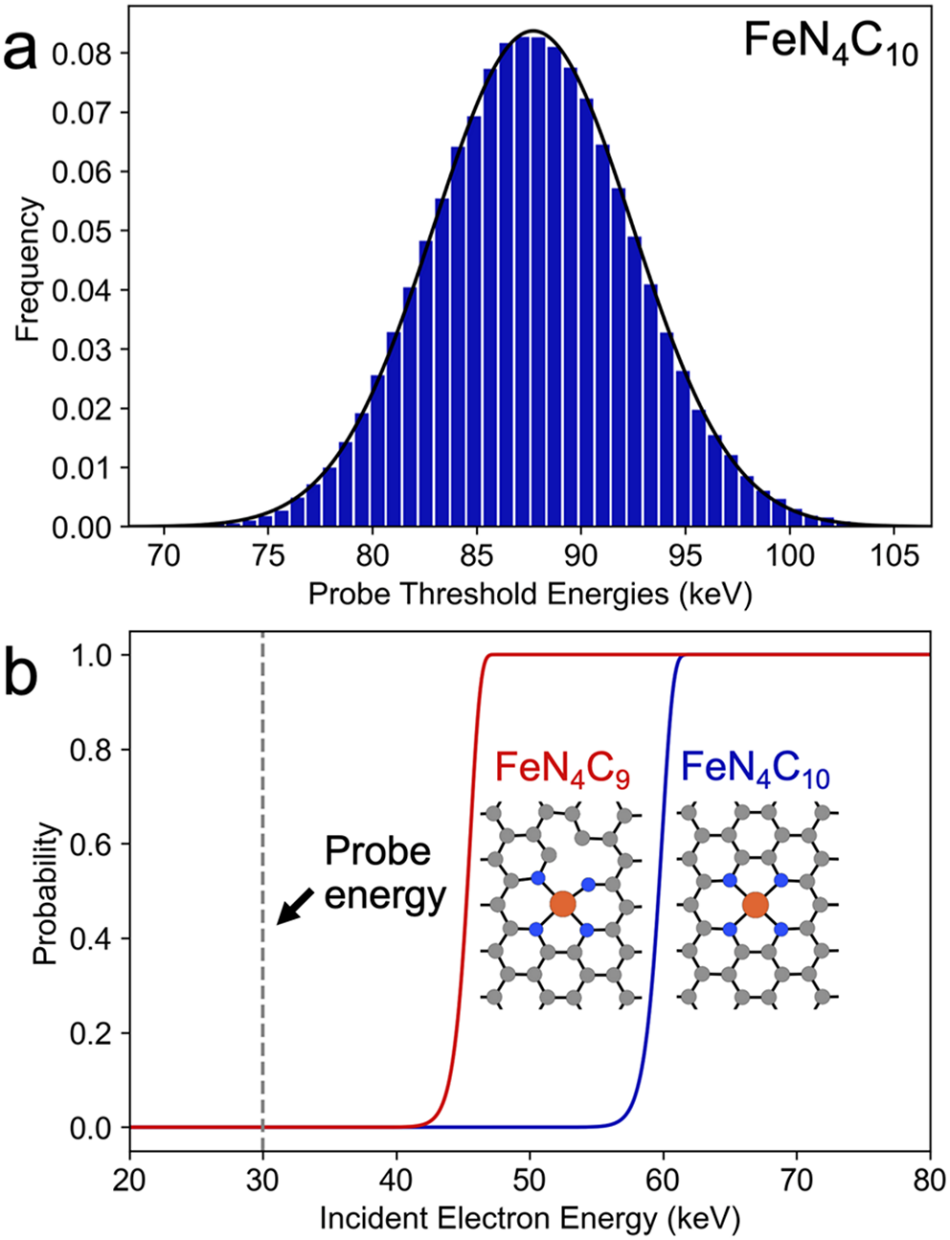
Effect of electron probe energy on damage probabilities and attainable resolution. (a) Example of knock-on displacement electron beam energy threshold (EBET) calculated for a FeN_4_C_10_ structure at 300 K by combined AIMD-DFTBMD, demonstrating broadening of the EBET due to thermal motions. (b) Cumulative probability of knock-on damage occurring within a single acquisition for ideal FeN_4_C_10_ and defective FeN_4_C_10_ (or FeN_4_C_9_) structures, utilizing calculations such as those in panel (a). The 30 keV probe energy used in the experimental work here, indicated by the dashed vertical line, minimizes structural modifications during acquisition.

While we calculated that this specific structure should be essentially stable for probe energies below approximately 55 keV, other site structures and local defects could have lower EBETs, requiring lower probe energies to minimize structural modifications.^31^ Figure 3b shows, for example, the effect of a local defect on the stability of an FeN_4_C_10_ site (represented by FeN_4_C_9_). In this case, a single missing carbon atom near the metal site reduces the damage threshold by more than 15 keV, requiring probe energies below approximately 40 keV for stable imaging. Since these thresholds correspond to full displacement of an atom from a structure and less energy may be still be able to rearrange atoms within a structure,^46,47^ we utilized a specialized instrument capable of generating a 30 keV electron probe, which is more typical of a scanning electron microscope (SEM) than a STEM, to minimize modifications to the sample. Under these ultralow voltage operating conditions, incident electrons are predicted to have nearly zero probability of causing knock-on damage during an acquisition, even for sites with neighboring defects, nearly independently of reasonable probe currents or applied doses.

While this reduced probe energy minimizes knock-on damage, the trade-off is an increased incident electron wavelength, which reduces attainable spatial resolution. Figure S6 demonstrates this effect using multislice electron scattering calculations. These results show, however, that with an optimized experiment, the atomic-scale lattice structure around a site should still be accessible at 30 keV. While neighboring carbon atoms are no longer clearly resolved in this case, their locations can be inferred, given their presence at the vertices of the positive contrast features formed by the lattice structure. The calculations shown in Figure S6 represent approximately ideal experimental results, however, and residual probe aberrations and noise can further degrade the ability to resolve structures in real experiments. Due to the image formation process, dCoM images such as those shown in Figure 2 further amplify high-spatial-frequency noise,^41^ which can complicate interpretation of atomic-scale contrast, especially for low electron doses or when structures are near the diffraction-limited resolution of the instrument. Although spatial filters can be applied to suppress noise at frequencies beyond the information limit of the instrument, residual aberrations and remaining noise still make accurate determination of lattice structure challenging for any filter size (Figure S1). Figure S7a,b shows, for example, experimental ADF and dCoM images, respectively, acquired with a 30 keV probe. As expected, ADF carries minimal information about the carbon structure, while dCoM reveals both the metal site and information about the surrounding lattice. However, accurate identification of lattice atom positions remains challenging in the dCoM image due to noise, despite the application of a low-pass Gaussian filter.

To enable the atomic-scale structure around active sites to be accurately determined using a 30 keV probe, we therefore utilized an alternative 3D-STEM technique, electron ptychography, which can reconstruct the phase change of the electron probe induced by the sample. This results in a contrast similar to dCoM (though the object function is proportional to the projected potential of the sample, like iCoM) with an improved signal-to-noise ratio (SNR) and the ability to minimize residual probe aberrations.^48–55^ To facilitate this ultralow-voltage ptychography, our 30 keV instrument was equipped with a cold field-emission gun (FEG) to minimize the energy spread of the probe and hence the effects of chromatic aberrations, which can dominate at low voltages. The 30 keV probe and cold FEG were paired with a pixelated direct electron detector that has an optimal performance at low beam energies^56^ to maximize data quality. Furthermore, we explored and optimized various forms of electron ptychography, with aberration-corrected Wigner distribution deconvolution providing the optimal results for our data at 30 keV, and optimized the experimental parameters for the beam energy, technique, and material used, such as tuning the probe convergence angle so that the maximum of the contrast transfer function coincided approximately with the spatial frequencies of the atomic lattice in our material. A comparison of results from a selection of ptychography methods is shown in Figure S10, and examples of parameters varied to optimize the final aberration-corrected Wigner distribution deconvolution results are shown in Figures S11–S13.

Figure S7c shows the resulting electron ptychography phase reconstruction that corresponds to the ADF and dCoM results in Figures S7a,b, and the improved SNR is further explored and quantified in Figure S8. With probe aberrations minimized and SNR improved (without any postprocessing spatial filters applied for noise suppression), the ability to identify atomic sites in the graphitic lattice is substantially improved. As predicted, the 30 keV probe also allows this information to be obtained while minimizing alterations to the structure within the graphitic lattice, as shown in Figure S7. This is true even for metal sites containing neighboring defects, such as the structure calculated in Figure 3b, as shown in Figure S9. This is significant, as these sites play an important role in determining catalyst activity and stability,^20^ therefore, accurate characterization of their structure is essential. While a 30 keV probe essentially eliminates knock-on damage, it is worth noting that the edges of graphitic basal planes can slowly etch in STEM instruments that do not operate at ultrahigh vacuum (UHV), likely due to interaction of trace molecules in the vacuum with the beam and subsequently the sample.^57^ Though metal sites and atomic-scale lattice defects are not initially affected by this process, extended exposure can result cause sample edges to interfere with these structures (Figure S9). For a typical sample area and acquisition, such as that shown in Figure S9, the edges of the sample recede by roughly 2 Å per second of data acquisition. For areas with as little as 1 nm of surrounding material, this could limit total acquisition times to minutes. However, for areas with large distances to graphitic basal plane edges, this time could be significantly longer. Use of a UHV instrument could increase these times by an order of magnitude or more as well.

Using this 30 keV electron ptychography technique, the native structure of individual CMS active sites can be directly obtained experimentally, compared to idealized predicted structures, and used to understand overall catalyst properties.^18^ Figure 4 compares two commonly proposed idealized active site structures to a site structure experimentally observed in our well-defined CMS catalyst by 30 keV electron ptychography. Figure 4a,b shows multislice electron scattering calculations for two commonly proposed active site structures, FeN_4_C_10_ and FeN_4_C_12_,^10–12,23^ with the local lattice structure identified; white dots indicate lattice atom positions surrounding the metal site, green fill indicates six-membered graphitic rings, blue fill indicates five-membered rings, and gray fill indicates larger voids. Given structural models like these, computational methods can be utilized to calculate the properties of the sites, such as their activity and stability. For example, since local corrosion of the lattice around CMS catalyst active sites and the resulting degradation is often kinetically limited,^58–60^ the computational framework that allowed us to calculate EBETs can be utilized to provide a description of the stability of sites with respect to carbon/nitrogen corrosion,^19,20,31,44,45^ which is a significant proposed degradation mechanism for these catalysts. Thus, far, however, calculation of predicted CMS catalyst active site properties has relied on idealized structures provided by characterization techniques that average over large numbers of sites, such as the symmetrical sites in Figure 4a,b. Thirty keV electron ptychography allows individual active site structures to be directly observed, therefore providing an understanding of how these structures may vary from their idealized counterparts.

**Figure 4:**
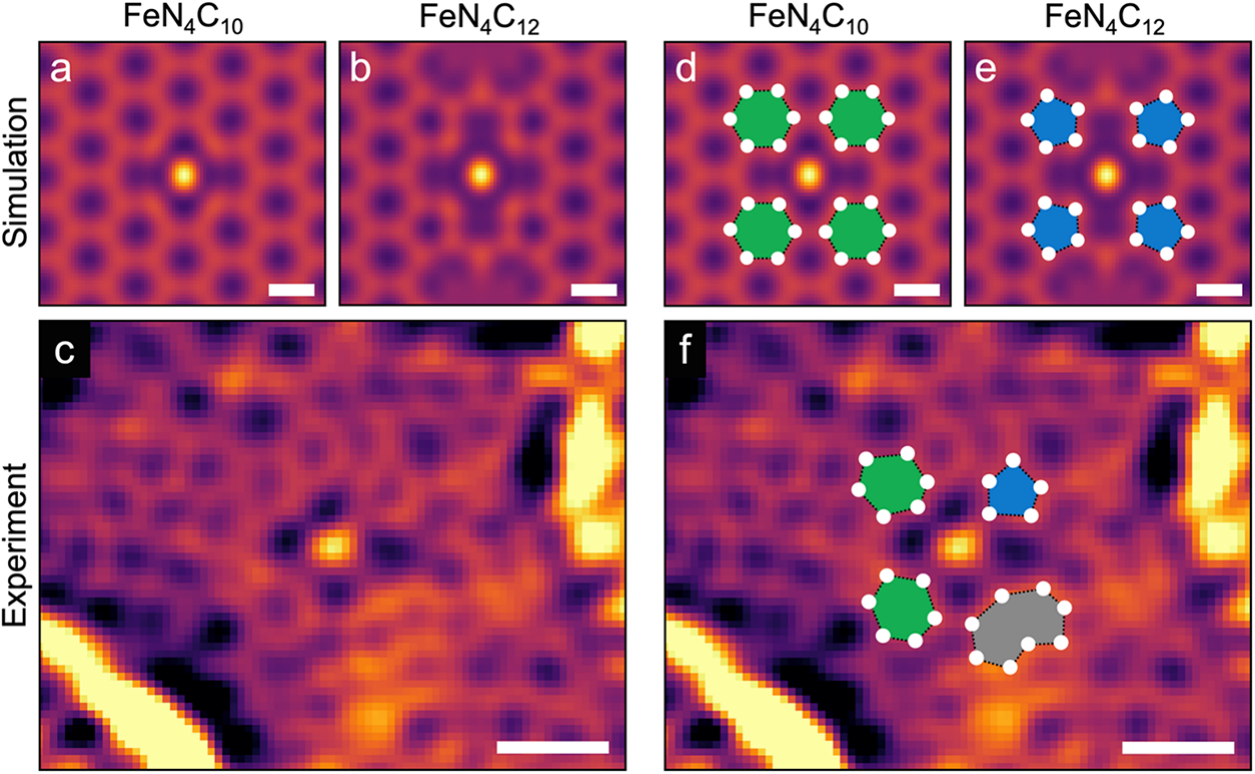
Comparison of individual active site structure experimentally observed by 30 keV electron ptychography and ideal structures. (a, b) Simulated results for idealized FeN_4_C_10_ and FeN_4_C_12_ proposed active site structures by multislice electron scattering calculations, and (c), experimental CMS active site structure observed by 30 keV electron ptychography. (d, e) Simulated structures from (a, b) with white dots labeling atomic sites within rings neighboring the metal atom, blue fill for five-member rings, green fill for six-member rings, and gray fill for eight-member rings. Differences in local structure, such as between these two site types, play a significant role in the performance and stability of active sites. (f) Experimental results labeled as in (d, e), highlighting how the structure neighboring the metal atom at individual sites in real materials can vary significantly from predicted structures, and as a result, their properties could vary correspondingly. Scale bars, 2 Å (a,b) and 5 Å (c).

Figure 4c shows an example of an experimentally observed individual site structure in our well-defined CMS catalyst that contains a mixture of randomly positioned five- and six-membered rings around the metal site, as well as a more complex structure, as opposed to the symmetrical nature of the idealized sites in Figure 4a,b. As a result of the observed nonsymmetrical, unconventional structure, the properties of this site could vary significantly from either of these symmetrical structures. To demonstrate the possibility of pairing direct, experimentally observed site structures with calculations of individual site properties, we explored the stability of site structures similar to that observed in Figure 4 and performed calculations of properties relevant to the oxygen reduction reaction (ORR) by DFT. Figure S14 shows a stable structure found to recreate well the arrangement of rings adjacent to the metal site, which largely dictates the electronic structure and ORR properties of the site, which included a five-membered ring, two six-membered rings, and, interestingly, an eight-membered ring.

Our calculations show that such a site strongly overbinds ORR intermediate species, yielding a value of 0.14 V vs CHE, with the last protonation step being potential-determining. This structure is a strong candidate for spontaneous ligation due to this overbinding, as well as local defects, which may provide easier access to the axial fifth position without blocking surface reactivity. We have therefore found not only that sites with nonideal structures are present in CMS materials, even those with well-controlled structures, but also that the electrochemical properties of these sites can be far from optimal and thus need to be controlled, which will be the focus of future work. In addition, this demonstrates how 30 keV electron ptychography provides a pathway to accessing information about CMS sites that was previously unavailable, allowing direct experimental observations of local CMS site structures to be coupled with modeling to generate an understanding of the impact of the variety of sites and corresponding properties actually present in CMS materials.

While these techniques will immediately enable atomic-scale insights into a variety of thin, electronically conductive materials, such as those about local structure around dopants or defects in conductive 2D quantum materials, they may be further developed in the future to provide enhanced information about sites. For example, advanced ptychographic methods could be utilized to reduce chromatic blurring and improve image resolution beyond the diffraction limit, enabling atom positions to be more precisely measured.^32,33,53,55,61^ Furthermore, the method utilized here is limited to generating 2D projections of a 3D structure, complicating analysis of thicker samples. More advanced ptychography techniques may also be able to provide depth resolution, allowing atomic positions to be measured in three dimensions as well as allowing thicker materials to be characterized accurately. The ability for these techniques to provide real-space upsampling, in addition to development of faster detectors, will significantly decrease total data set acquisition times and hence allow increased throughput. Combined with agentic, autonomous experiments that can intelligently target acquisition locations, this will enable significantly more robust statistics about local site structures to be obtained. Combined with high-throughput, AI-based computational techniques to provide information about the electronic structure and activity of vast numbers of unique, individually measured sites, this will make ptychography a compelling alternative to electrochemical scanning tunneling microscopy^62–64^ for providing individual active site structures and activities, as well as the effect of their local environments on, for example, overpotentials due to mass transport limitations, when active sites are away from surfaces. For straightforward experiments with enhanced resolution, improved aberration correction, and chromatic aberration correction could be combined with direct ptychographic methods like those demonstrated here. Low-dose and cryogenic techniques may, furthermore, enable increased probe energies to be used while minimizing structural modifications (Figure S15), leading to enhanced spatial resolution. Atom-finding computational methods could rapidly identify atom positions,^65^ increasing the throughput of the technique and decreasing the subjectivity of the results. Combined ptychographic-tomographic and multislice ptychographic techniques could also provide out-of-plane structural information about the sites.^51,55,66–69^ Each of these techniques could be performed in a UHV instrument to minimize etching,^57^ further reducing lattice motion and extending the time that a site can be imaged, possibly allowing *in situ* observations over long time scales, improving data quality, and enabling sites at edges of the material to be more accurately characterized. Paired with the structural information provided by ptychography, performing EELS at ultralow voltages would allow extended measurements with enhanced statistics, enabling the local bonding environment, and hence coordination, of each site to be confidently inferred. Finally, suppressing structural modifications could enable identical location studies^70^ to be performed, where individual sites are examined in a pristine state and subsequently after cycling to provide insights into the structural degradation mechanisms. In each of these cases, techniques for autonomous metal atom finding and data acquisition^71^ would allow a wide variety of sites to be characterized, building a more complete picture of the connection between the atomic-scale structure and properties in these materials.

## Conclusion

Single-atom CMS electrocatalysts are promising next-generation catalysts for a wide range of applications. Understanding their activity, selectivity, and stability of these materials requires an understanding of both their average properties across length scales and the atomic-scale variations in local site structures. Thus, far, however, reliable local measurements of atomic-scale site structures were not possible by high-resolution electron microscopy due to the highly sensitive nature of the sites with respect to high-energy electrons. In this work, we demonstrated ultralow-voltage electron ptychography using a 30 keV electron probe, which minimized probe-induced damage and allowed the atomic-scale structure of individual CMS sites to be directly imaged for the first time. Further advancements in electron ptychography may allow additional information to be obtained from these sites, including in three dimensions, and autonomous experiments may allow vast numbers of site structures to be measured using these techniques. As a result, the work shown here will serve as a springboard toward a comprehensive understanding of the breadth of individual CMS active site structures present in CMS catalysts and their degradation mechanisms, which, when paired with computational techniques, will accelerate the design of a range of next-generation single-atom catalysts important for achieving a sustainable future.

## Methods

### Sample Synthesis and Electrochemical Analysis

Dopamine hydrochloride (Sigma-Aldrich) was dissolved in Tris-HCl buffer solution (pH 8.5) in the presence of FeCl_3_•6H_2_O, followed by the addition of NaCl (Sigma-Aldrich) as a template for the formation of a thin two-dimensional graphene structure. The resulting mixture was dried in a vacuum oven overnight, followed by heat treatment under Ar gas at 700 °C for 2 h in a tube furnace. The resultant carbon powder was thoroughly washed with deionized water (DI water) several times to remove the NaCl. After vacuum treatment at 70 °C overnight, a second heat treatment under NH_3_ gas was conducted at 900 °C for 15 min, producing a material denoted as Fe–N–C. For comparison, a parallel experiment was conducted without the addition of Fe salt to produce a metal-free electrocatalyst, designated as N–C. Electrochemical oxygen reduction reaction (ORR) was analyzed under a three-electrode system using a PGSTAT 302 (Metrohm Autolab). The glassy carbon rod and an Ag/AgCl electrode were used for the counter and reference electrode, respectively. The glassy carbon working electrode 0.283 cm^2^ was used. All potentials are reported relative to the reversible hydrogen electrode (RHE) after correction of each measurement. The catalyst loading of carbon-based materials was fixed by 600 μg cm^–2^. For comparison, commercial Pt (TKK 46 wt %) was also analyzed (20 μg_Pt_ cm^–2^). The ORR activity was measured under the O_2_ saturated electrolyte with rotation speed of 1600 rpm and a scan rate of 10 mV s^–1^. Non-Faradaic capacitive current and solution resistance were corrected for the report.

### STEM Data Acquisition

Data for Figure 4 and Figures S1, S4 (30 keV data and a portion of the 60 keV data), and S7–S13 were acquired on a JEOL NEOARM aberration-correction scanning transmission electron microscope (STEM) operated at an accelerating voltage of 30 kV, a semiconvergence angle of ∼28–35 mrad, and a probe current of ∼25–40 pA. Data was collected on a pnCCD pixelated detector with minimum collection angles (narrowest part of square detector) between 37 and 65 mrad and a dwell time of 1 ms per pixel. For Figure S2, the same instrument was used with an accelerating voltage of 60 kV and data were collected on a Gatan 965 Quantum ER GIF. Data for Figure 2 and a portion of the 60 keV data in Figure S4 were acquired on a Hamamatsu ORCA CMOS detector in a Nion UltraSTEM 100. The instrument was operated at an accelerating voltage of 60 kV, with a probe current of ∼ 50 pA, a dwell time of 4 ms per pixel, and a minimum collection angle of ∼40 mrad. The vacuum in the JEOL NEOARM is typically <10^–7^ Torr, while the vacuum in the Nion UltraSTEM 100 is typically ∼10^–8^ Torr.

### Data Analysis, Multislice Calculations, and Visualization

To perform the center of mass (CoM) analysis and visualize the results, custom Python codes were used that follow techniques reported previously.^40,41,72,73^ Briefly, the CoM of the diffraction pattern acquired at each probe position was calculated, and the dCoM images were produced by taking the divergence of these CoM measurements by taking derivatives through Fourier space. Ptychography was performed using a Wigner Distribution Deconvolution (WDD) algorithm in the PtychoSTEM MATLAB package,^74^ following methods that have been discussed previously.^48,51,54^ WDD involves calculation of the sample Wigner distribution function through a series of fast Fourier and inverse Fourier transforms with respect to multiple variables and a subsequent Wiener deconvolution. The complex sample transmission function can then be determined, which provides information about the phase change of the electron beam due to the interaction with the sample and hence the projected potential of the sample. Residual aberrations can also be determined using this technique through singular value decomposition,^51^ for example, and used during the deconvolution to minimize their effect on results. In this case, we found an epsilon value around 1.0 optimized the results, as shown in Figure S11. The probe spacing also had to be matched to the experimental value accurately, with even ± 0.2 Å beginning to degrade contrast, as shown in Figure S12. Finally, around ten iterations of aberration determination maximized the contrast, and hence signal-to-noise ratio of the results, as shown in Figure S13. Optimizing these values is essential for ensuring the projected potential is most accurately reconstructed and hence maximizing the fidelity of structural determinations, as can be observed in these figures. Comparison of these results with appropriate simulations, such as those in Figures 1, 4, and S6, of various structures is also important for proper interpretation of the results. For consistency, the ptychographic results were visualized using the same Python codes used to visualize the dCoM results.

To calculate simulated images, electron multislice calculations were performed in Python using pyQSTEM^75^ (Figure 1) and abTEM^76^ (Figure 4). Beam parameters were chosen to match those of the experiments, and no probe aberrations or focal spreading were included. The pyQSTEM results were analyzed and visualized by using the same Python codes as the experimental data. The abTEM CoM values were determined using its built-in CoM functionality, and the same Python codes used to analyze the experimental data were subsequently used to calculate the dCoM image from these results and visualize the results.

Beam parameters were chosen to match the experimental conditions, but no probe aberrations or focal spread was included. Results were analyzed using the same Python codes as those used for the experimental data analysis.

### Electron Beam Energy Threshold Calculations

The EBET of N atoms embedded in various proposed FeN_4_C_*x*_-based CMS active site structures are calculated using our AIMD-DFTBMD approach to model electron beam damage in transmission electron microscopy (TEM).^19,43^ The EBET captures the kinetics of bond breaking in an atomic structure by estimating the minimum electron beam energy needed to liberate an atom from its bound state by knock-on processes.^31^ To accurately predict the range of EBET values at finite temperatures, we combined ab initio molecular dynamics (AIMD) and density-functional tight-binding (DFTB)-based MD simulations. Our method consists of a two-step approach. First, we perform AIMD simulations to calculate the EBET as a function of the perpendicular displacement of a N atom embedded in a given atomic structure. Single-layer 6 × 6 structures are considered to model the basal plane host configuration, with a vacuum spacing in the direction perpendicular to the graphene plane of ∼15 Å. We consider generalized gradient approximation in the form of Perdew–Burke–Ernzerhof’s exchange-correlation functional,^77,78^ as implemented in the Vienna ab initio simulation package (VASP).^79–82^ The initial geometries prior to molecular displacement are optimized self-consistently using density functional theory (DFT) with cell shape fixed to that of bulk graphene (allowing ionic relaxations). Using these optimized FeN_4_C_*x*_ structures, we carry out a ∼150 fs AIMD simulation where a symmetry-equivalent N atom at rest is given an initial kinetic energy for various perpendicular z-displacements from the lattice. For a given z-displacement, we consider the lowest applied beam energy (in a range of values separated by 1 keV) that is large enough to eject a N atom *ca*. 4 Å from the lattice plane and create an on-site vacancy in the lattice to be the associated 0 K EBET value. A time step of 0.5 ps is chosen which gives an accurate prediction of the dynamics, as previously reported by Su et al.^47^ All calculations consider spin polarization, a plane wave cutoff energy of 560 eV, and a γ point k-mesh. In addition, the effects of van der Waals interactions are introduced by dispersion forces using the DFT-D2 method of Grimme,^83^ and dipole correction along the *z*-direction is assumed to avoid spurious interactions between repeated images. The self-consistent electron density loop is converged to 10^–5^ eV for AIMD and structural relaxations, and the ionic relaxation loop is run until calculated forces are less than 0.02 eV/Å for structural relaxations. This process is repeated with displacements of the N atom out of plane up to 1.75 Å to derive an EBET vs atomic displacement relation to be used for calculating temperature-dependent EBET distributions.

Next, we perform a temperature correction to obtain EBET values as well as corresponding threshold probe energies considering knock-on displacement elastic scattering of an electron from an atomic nucleus. In (quasi-)­elastic scattering, the maximum kinetic energy transfer to a target nucleus from an accelerating electron is possible only if the electron backscatters after the collision.^53^ For a backscattering event (the scattering angle $\theta =180^{{}^{\circ}})$ between an incident electron with kinetic (beam) energy $E_{d}$ and rest mass m and an atomic nucleus with mass M moving at velocity v parallel to the beam, the elastic energy transferred from the electron to the nucleus can be expressed as,^43^

1 $${Ẽ}_{n}\left(E_{d},v\right)=\frac{{\left(2\sqrt{E_{d}\left(E_{d}+2mc^{2}\right)}+\mathrm{Mvc}\right)}^{2}}{2Mc^{2}}$$

where *c* is the speed of light. The AIMD-derived EBET values obtained above can be considered to calculate the minimum probe energy required to transfer sufficient elastic energy to an N atom at rest to displace it from the lattice. However, the temperature effects assume fluctuations from the rest position where the velocity term in (eq 1) contributes to the elastic energy transfer as a correction to the total kinetic energy. Therefore, EBETs should be smaller for atoms moving in the direction of incident electrons compared to atoms at rest and larger for those moving opposite to the incident electrons.

To account for this correction along with statistics of N displacements out of the graphene plane, DFTB-based MD simulations are performed on the optimized FeN_4_C_*x*_ structures to obtain displacement and velocity distribution of N atoms at temperatures *T* = 100, 300, and 500 K. We utilize the DFTB package implemented in Amsterdam Density Functional (ADF) modeling suite^84^ and consider a self-consistency loop for the Mulliken charges (SCC-DFTB). We use a time step of 0.2 fs and carry out 100 ps MD simulations at different temperatures with the Berendsen thermostat. The Slater-Koster parameters “trans3d-0–1” for transition metal elements^85^ are used as DFTB parameters, and a γ point is used to sample the Brillouin zone. The MD trajectories are collected with a sampling frequency of 10 (every 2 fs) and normal distributions of probabilities for the displacements and velocities are obtained at a temperature T based on these statistics. These displacement and velocity statistics as a function of temperature are used to determine the probability distribution for EBET following the AIMD-derived EBET vs displacement and velocity dependence described by eq 1. The corresponding probability distribution for EBET are then also determined as a function of temperature. This is then fit to a normal distribution such that the mean and standard deviation can be easily reported. These fits match obtained EBET distributions as shown in Figure 3a and serve as a way of describing the temperature-dependent threshold probe energy distribution using two parameters.

As described in the main text, we then combine the means and standard deviations of the EBET calculations with reasonable assumptions of instrument and sample parameters to generate a probability distribution for a knock-on event occurring within a given acquisition. Here, we matched the parameters to the experimental 4D-STEM settings that produced the maximum electron dose utilized for any data set, namely, a 50 pA probe current, a dwell time of 4 ms per probe position, 128 × 128 probe positions, a probe spacing of 3.30 Å, and a susceptible area of 150 Å^2^, estimated by adding the probe (estimated from experimental resolution) and susceptible atom sizes in quadrature and approximately doubling the result to provide a margin for error (the probability distributions are only weakly dependent on this value, however, with the energy that gives 50% probability of knock-on damage for FeN_4_C_10_ changing only by ∼ 5.5 keV for areas ranging 3 orders of magnitude from 1 Å^2^ to 1000 Å^2^).

Finally, to determine if transition metal type plays a significant role in EBET, the Fe in FeN_4_C_10_ was replaced with Co and Mn. Only a very minor shift in undisplaced EBET values for neighboring N were calculated, ∼1 and ∼ −1 keV vs Fe, respectively, likely due to small strain effects of the varied transition metal. Hence, C/N structure and thermal contributions are found to have much larger impact on EBET than metal species, suggesting the findings shown in this work are broadly applicable to M–N-C active sites with varied M speciation.

## Supplementary Material



## Data Availability

PtychoSTEM electron ptychography package is available at: https://gitlab.com/ptychoSTEM/ptychoSTEM.

## Notes

Spatial filter comparison, high-resolution elemental mapping, oxygen reduction reaction polarization curves, examples of site structures observed, temperature-dependent EBET calculations, probe energy dependent multislice image simulations, imaging technique comparisons and signal-to-noise analysis, size-dependent structural damage analysis, comparison of ptychography methods and parameter optimization, DFT-calculated defective site structure, calculated site damage probability as a function of temperature, electron dose, and site type, and table of acquisition and analysis parameters (PDF)

## Notes

Hasnain Hafiz: Battery Research and Development, General Motors, Warren, Michigan 48092, United States
