# Supplementary material for: Insights into Native Single-Atom Electrocatalyst Site Structures
Source: ACS Nano. 2026 Jun 6;20(24):17490–500. doi: 10.1021/acsnano.6c03527 (PMC13296601; doi:10.1021/acsnano.6c03527)
Supplement: Supplementary file 1 [file nn6c03527_si_001.pdf]

Supporting Information

Insights into native single-atom electrocatalyst site structures

Michael J. Zachman[1]*, Hasnain Hafiz[2,†], Colum M. O'Leary[3], Chaewon Lim[4], Dong Young Chung[5,6], Sirui Li[2], Subin Park[7], Jiheon Kim[7], David A. Cullen[1], Edward F. Holby[8], Vojislav R. Stamenkovic[4,5,9]*

[1]Center for Nanophase Materials Sciences, Oak Ridge National Laboratory, Oak Ridge, TN 37831, USA.
[2]Theoretical Division, Los Alamos National Laboratory, Los Alamos, NM 87545, USA.
[3]Department of Physics and Astronomy and California NanoSystems Institute, University of California, Los Angeles, Los Angeles, CA 90095, USA.
[4]Department of Chemical and Biomolecular Engineering, University of California, Irvine, CA 92697, USA.
[5]Materials Science Division, Argonne National Laboratory, Argonne, IL 60439, USA.
[6]Department of Chemical and Biomolecular Engineering, Korea Advanced Institute of Science and Technology (KAIST), Daejeon, 34141, Republic of Korea.
[7]School of Chemical and Biological Engineering, Seoul National University (SNU), Seoul, 08826, Republic of Korea.
[8]Sigma Division, Los Alamos National Laboratory, Los Alamos, NM 87545, USA.
[9]Department of Chemistry, University of California, Irvine, CA 92697, USA.
[†]Present Address: General Motors Global R&D Center, Warren, MI 48092, USA.

*e-mail: zachmanmj@ornl.gov, vrstamen@uci.edu

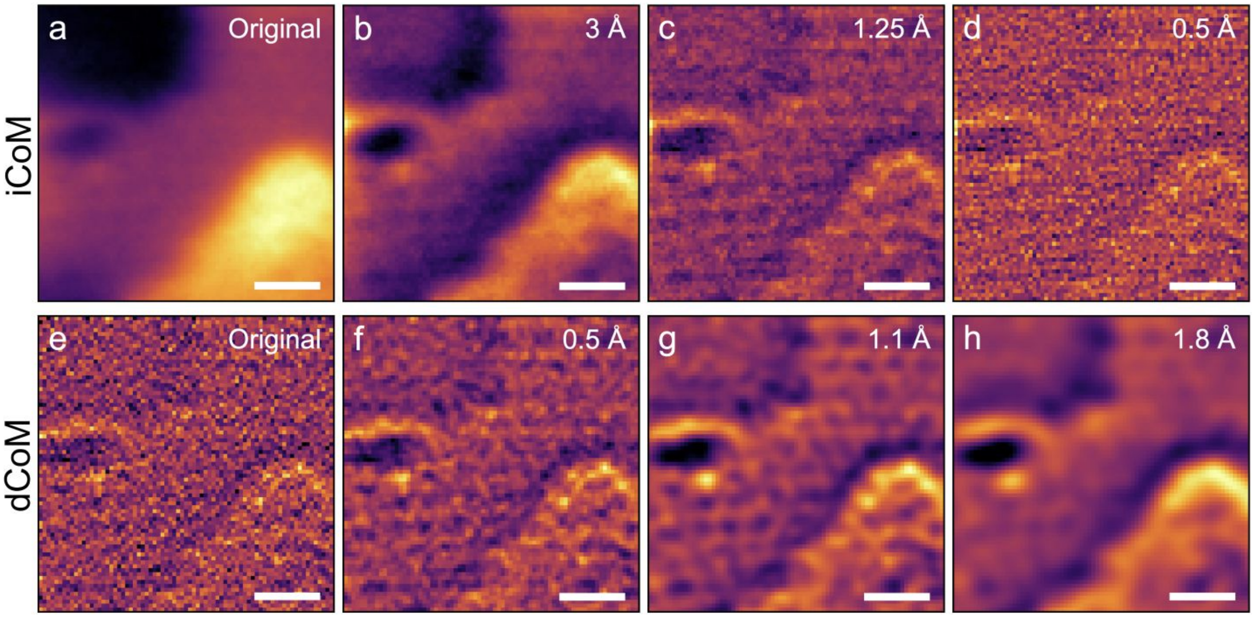

Figure S1. 30 keV iCoM and dCoM imaging with various spatial filters applied. (a)–(d) iCoM results for the data shown in Figure 2 of the main text with low frequency information suppressed by subtraction of Gaussian filtered images from the original. The full-width half-maximum (FWHM) of the Gaussian filter used is given on each image. (e)–(h) dCoM results for the data shown in Figure 2 with high frequency information suppressed by Gaussian filtering. The FWHM of the Gaussian filter used is given on each image. The dCoM image in (g) produces the optimal atomic-scale contrast and is shown in Figure 2. If the iCoM image in (c) is additionally Gaussian filtered to suppress high-frequency information, a similar result can be produced. dCoM therefore more directly provides the optimal atomic-scale contrast, in this case. Electron ptychography produces an image more well-suited than either iCoM or dCoM for identifying atomic-scale structure around the active sites, however, as shown in Figure 2. Scale bars, 5 Å.

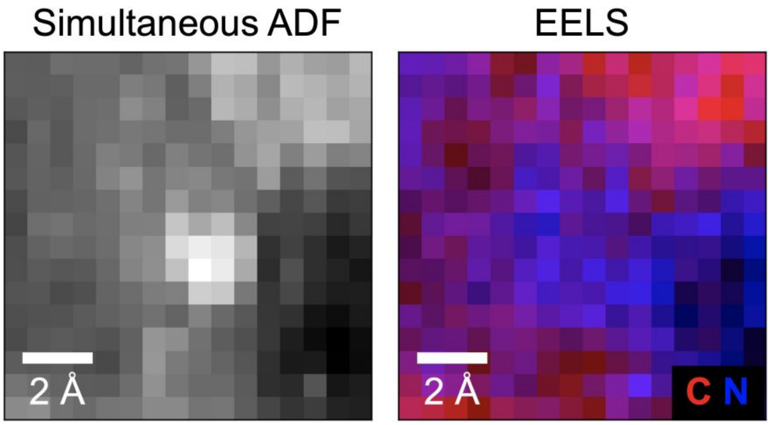

Figure S2. High-resolution elemental mapping around a PGM-free catalyst metal site by STEM-EELS. While annular dark-field STEM imaging (left) can reveal the location of heavier metal atoms, EELS mapping (right) can demonstrate the local presence of lighter elements such as nitrogen around metal sites.

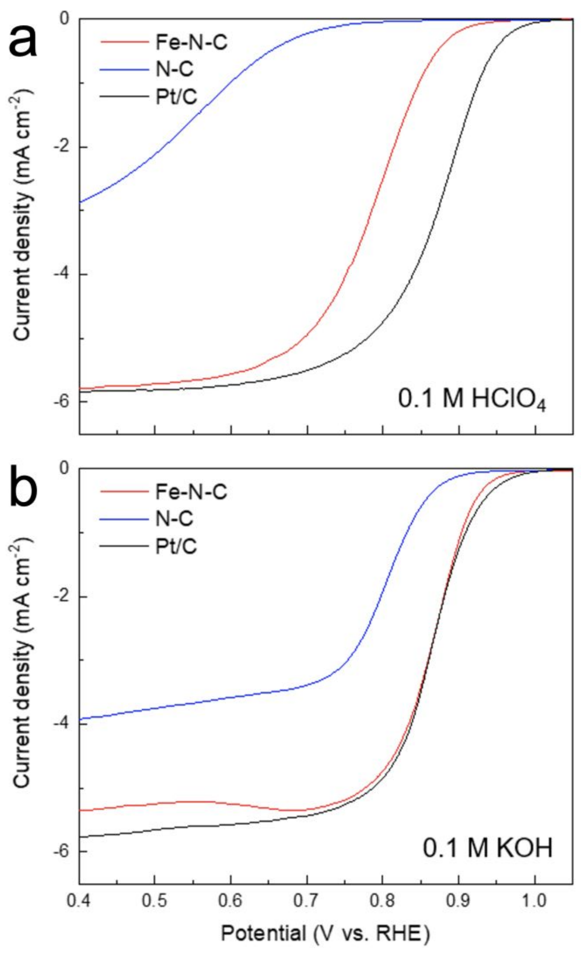

Figure S3. Well-defined CMS based on Fe embedded into graphene layer: (a) Electrochemical ORR polarization curves for Fe-N-C, N-C, and Pt/C catalysts in acidic environment (0.1 M $HClO_4$) and (b) alkaline environment (0.1 M KOH) with scan rates of 50 mV s^{-1} and rotation speeds of 1600 rpm. To provide a basis for comparison, the ORR activity of a Pt-based state-of-the-art system is also included.

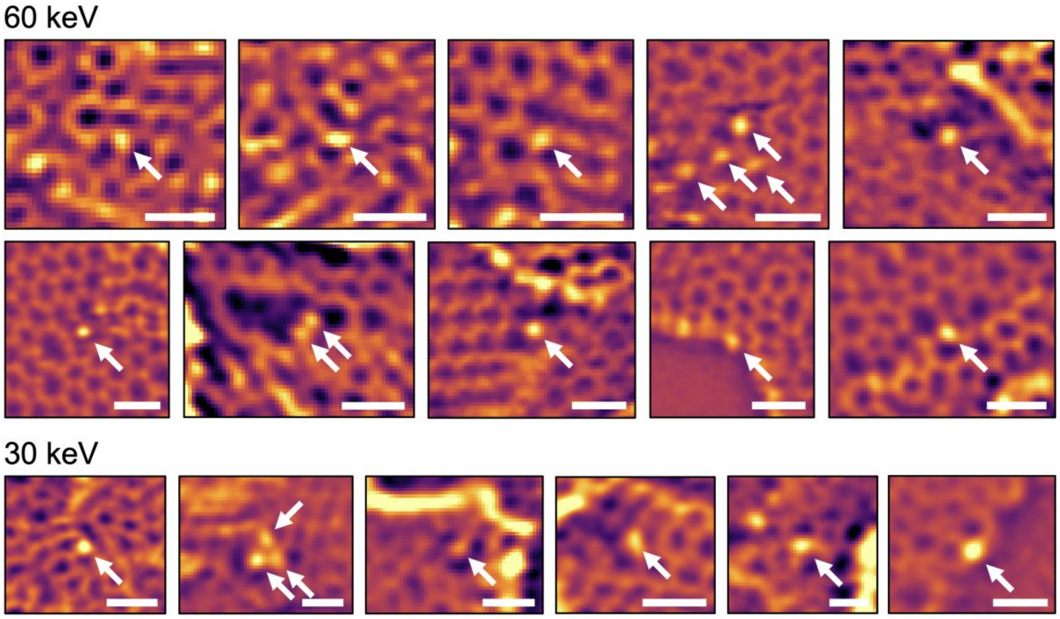

Figure S4. A selection of additional site structures observed both at 60 keV and 30 keV by high-throughput dCoM analysis. White arrows indicate Fe atom locations. As discussed in the main text, the structures observed at 60 keV may not be native to the material, since the beam energy is sufficient to modify the local structure of the sites. Scale bars, ~5 Å.

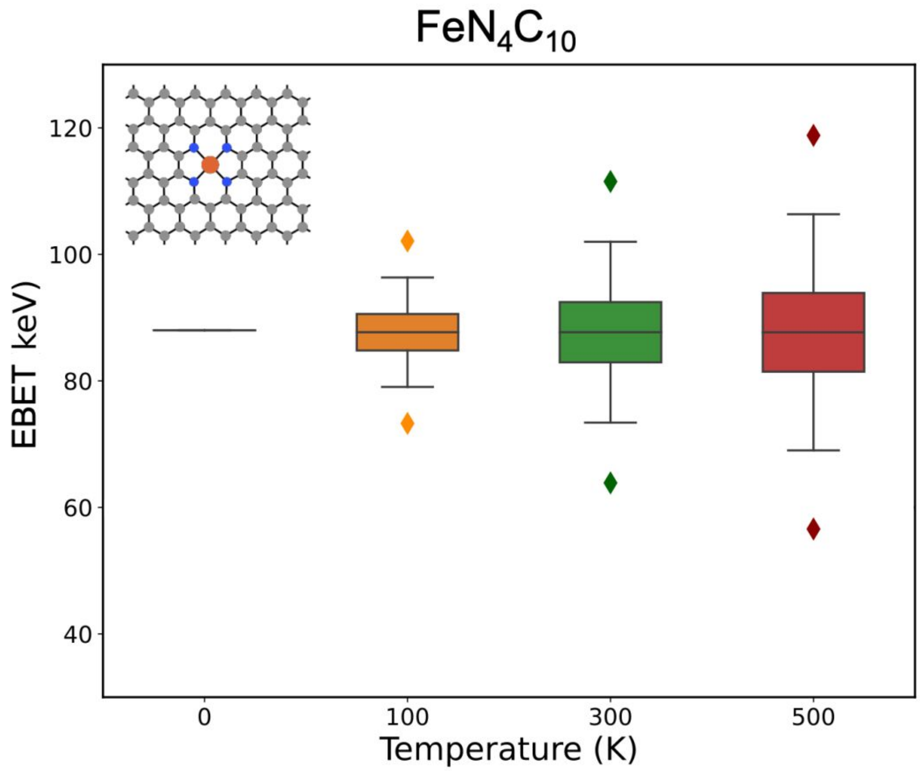

Figure S5. Temperature dependence of EBET distributions. For a static lattice, a given structure possesses a well-defined EBET, as shown here for an FeN_4C_{10} PGM-free catalyst site. At finite temperatures, this well-defined EBET becomes a distribution of damage probabilities due to the range of initial positions and velocities of the constituent atoms resulting from thermal motions, while the mean value is nearly independent of temperature. The width of the EBET probability distribution becomes larger with increasing temperature. In this plot, means of the distributions are displayed as solid horizontal lines in the colored boxes, and one, three, and five standard deviations are displayed as the bounds of the colored boxes, capped lines, and diamond markers, respectively.

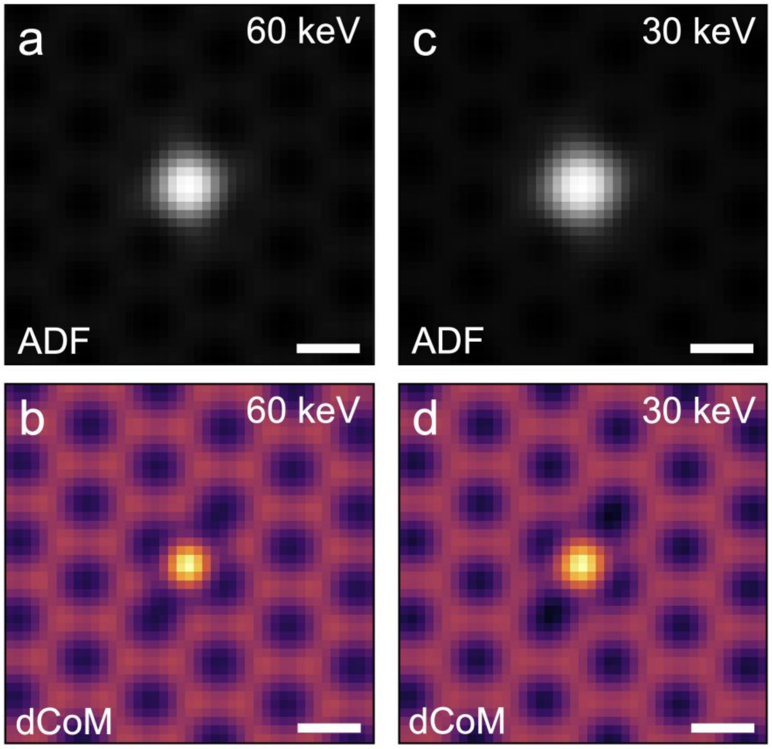

Figure S6. Effect of electron probe energy on attainable resolution. (a)–(b) ADF and dCoM multislice electron scattering calculations for an FeN_4C_{10} structure with a 60 keV probe. (c)–(d) ADF and dCoM multislice electron scattering calculations for the FeN_4C_{10} structure with a 30 keV probe, showing the reduced spatial resolution compared to the 60 keV case. Note that a ~0.5 Å Gaussian filter was applied in both cases to qualitatively approximate the appearance of experimental data, which is blurred, for example, by source-size broadening and residual aberrations. Scale bars, 2 Å.

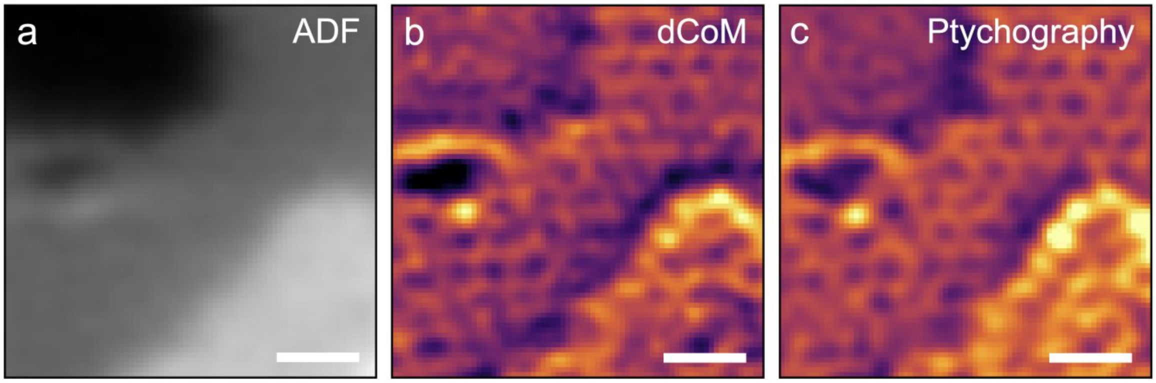

Figure S7. Comparison of ADF, CoM-STEM, and ptychography at 30 keV. (a) While ADF images provide little information about the carbon structure surrounding metal sites, (b) dCoM images are sensitive to this information at the atomic scale. Accurate identification of atomic sites is not always straightforward using dCoM, however, due to noise. (c) Electron ptychography phase reconstruction minimizes residual probe aberrations and generates images with improved SNR, providing a greater ability to estimate atom positions and hence accurately determine the atomic-scale carbon structure. Note that the ADF and dCoM images were Gaussian filtered here to remove noise significantly beyond the information limit of the instrument, while the ptychographic result was not filtered. Scale bars, 5 Å.

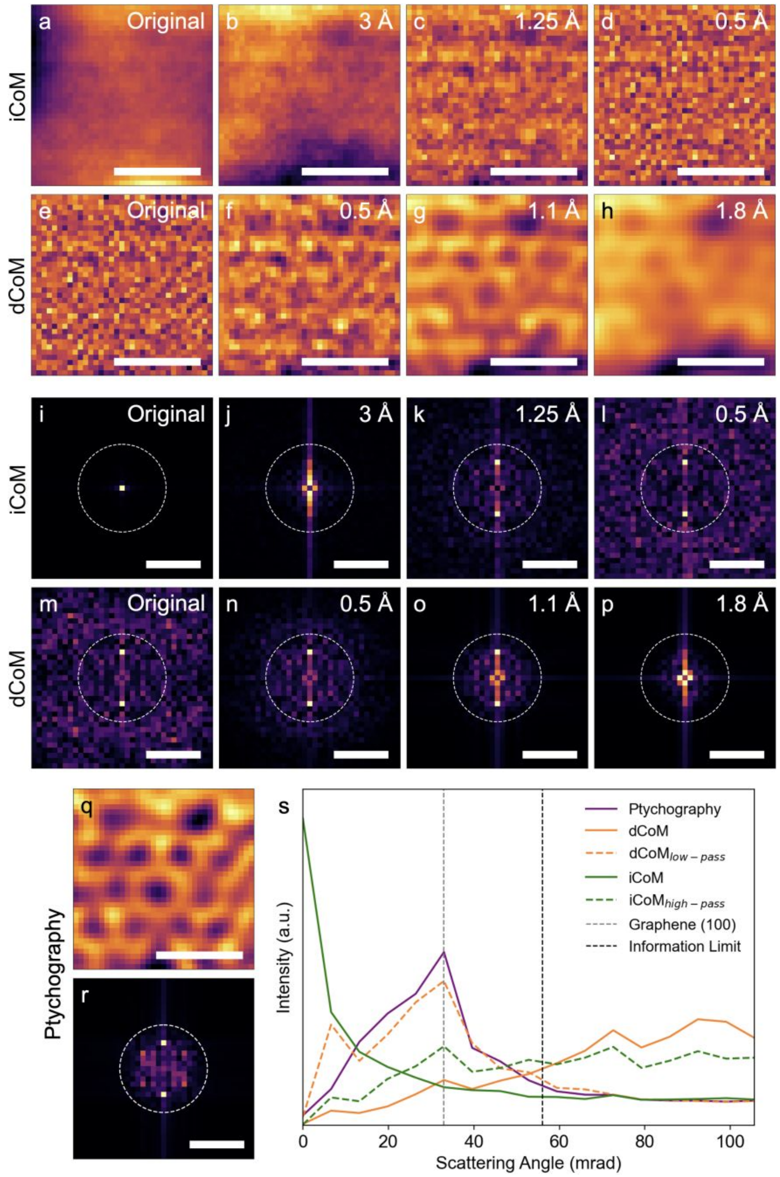

Figure S8. Signal-to-noise level comparison for filtered and unfiltered 30 keV iCoM, dCoM, and ptychography. (a)–(h) Unfiltered and filtered iCoM and dCoM results as in Figure S1 for zoomed region in top right showing monolayer graphitic plane. (i)–(p) Corresponding fast Fourier transforms (FFTs) of panels (a)–(h) showing the effect of the applied spatial filters on the iCoM and dCoM information transfer, with the instrument information transfer limit of twice the probe-forming aperture indicated by the white dashed circle. (q)–(r) Ptychographic analysis of the same dataset shows a higher signal-to-noise ratio (SNR) as indicated by the more well-defined lattice

structure in real space and higher Bragg peak levels compared to the background in reciprocal space. (s) Azimuthally summed radial FFT profiles of unfiltered and optimally filtered iCoM, dCoM, and ptychography. As a measure of SNR, the average signal at the graphene (100) Bragg peak frequency above that of the neighboring frequencies was divided by the summed intensities at frequencies above the information transfer limit. Ptychography produced the highest SNR; 1.8x higher than that of optimally filtered dCoM and 6.9x higher than optimally filtered iCoM. Each image separately normalized. Scale bars, (a)–(h), (q) 5 Å, (i)–(p), (r) 1 $Å^{-1}$ (70 mrad).

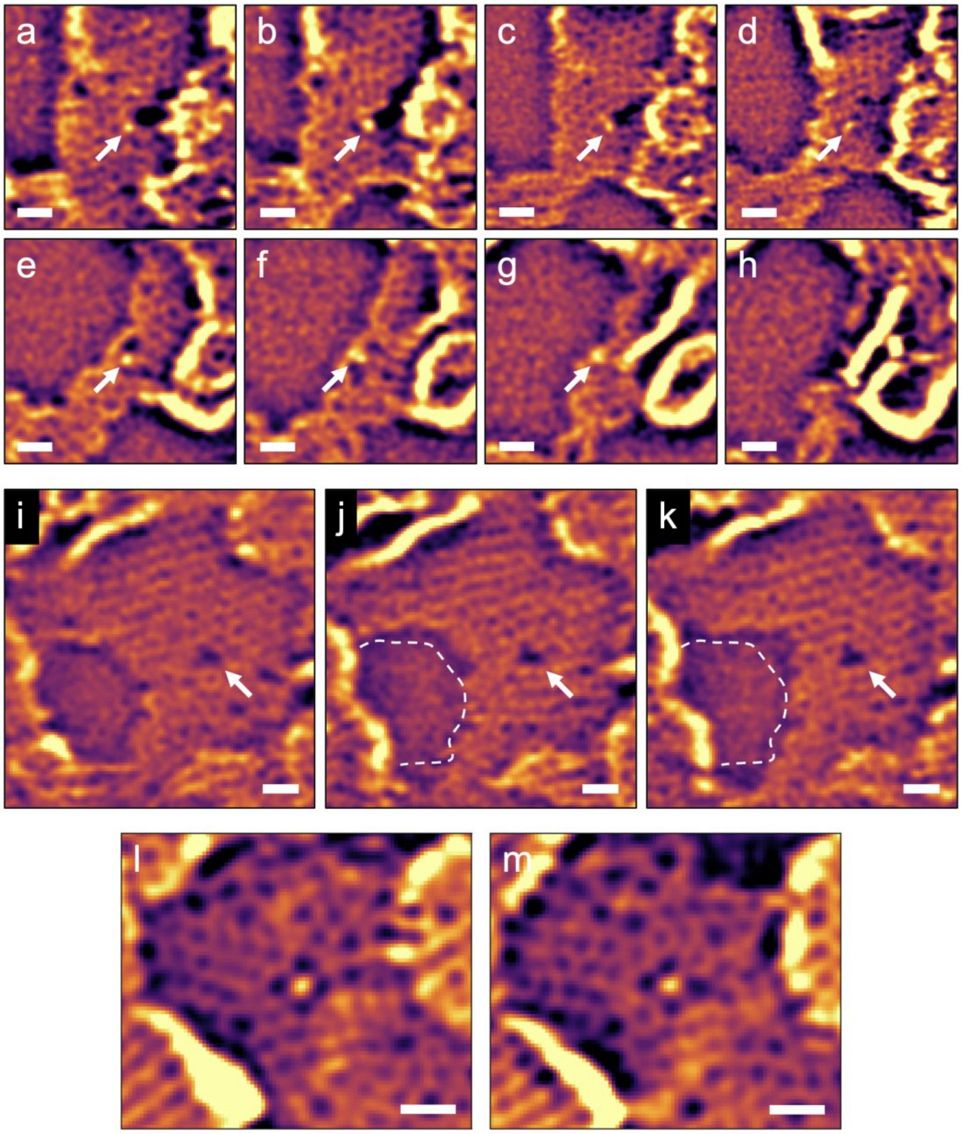

Figure S9. Stability of basal plane-hosted sites and effect of etching on basal plane edges and lattice defects when using a 30 keV electron probe. (a)–(h) Multiple acquisitions from a location in a PGM-free catalyst that includes a metal site (indicated by the arrows), neighboring lattice defect(s), and basal plane edges (displayed as dCoM images). While the metal site is initially stable when using a 30 keV probe, even with neighboring defect(s), etching of the basal plane edges occurs, eventually modifying the structure at the metal site. The time between the first and last exposure was approximately forty minutes. (i) 30 keV dCoM image of a large hole and a single missing carbon atom lattice defect (indicated by the arrow) in a PGM-free catalyst. (j)–(k) With

extended exposure, the basal plane edge of the large hole progressively receded (initial position indicated by the dashed line), while the single-atom defect was not affected. This shows that the etching process only occurs on extended basal plane edges and not at atomic-scale heterogeneities in the lattice. (l)–(m) A further example of the atomic-structure in the immediate vicinity of a metal site not changing significantly due to minimized knock-on damage at 30 keV, though etching of nearby basal plane edges occurs slowly and alters the strain within the lattice and correspondingly its structure. Scale bars, 5 Å.

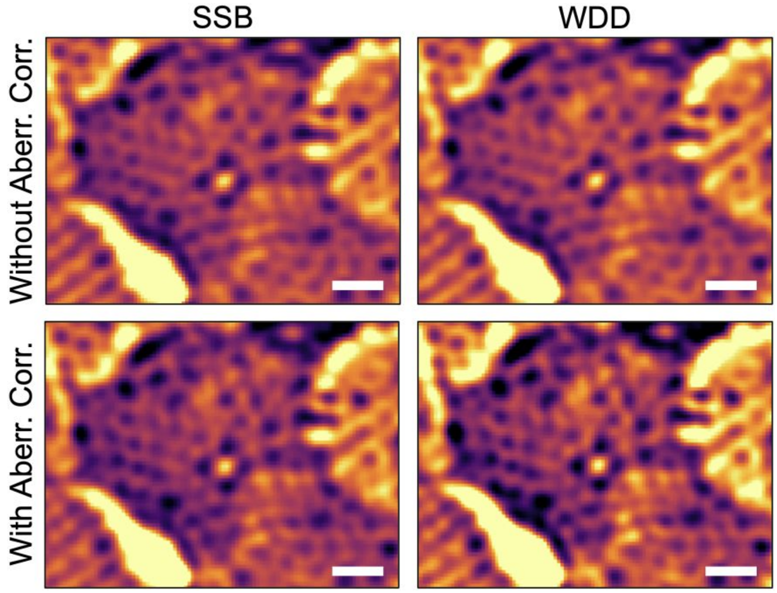

Figure S10. Comparison of optimized 30 keV single side band (SSB) and Wigner distribution deconvolution (WDD) results, with and without aberration correction included in the reconstruction process. Including aberration correction had the largest effect on WDD, increasing contrast and thus SNR (see Figure S8) more significantly than for SSB. Scale bars, 5 Å.

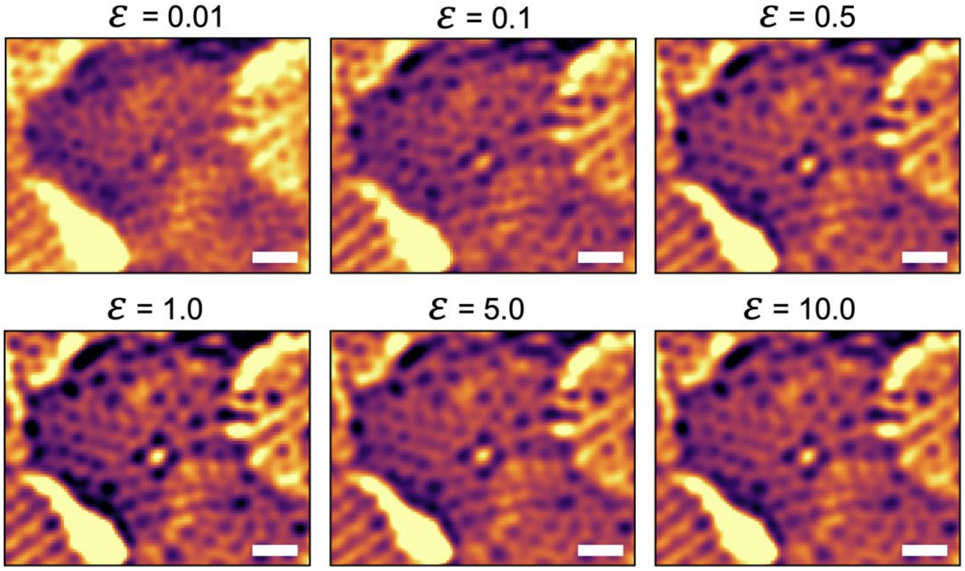

Figure S11. Comparison of ε values for 30 keV Wigner distribution deconvolution (WDD) results, with the optimal value being ~ 1.0 to maximize contrast and hence SNR (see Figure S8). Scale bars, 5 Å.

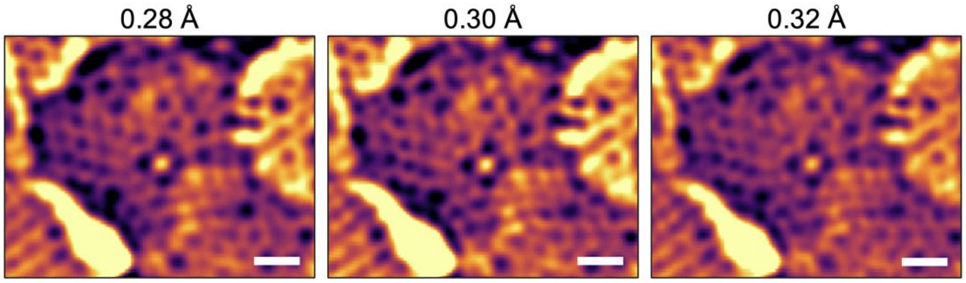

Figure S12. Comparison of different probe spacings utilized during analysis of 30 keV Wigner distribution deconvolution (WDD) results. The experimental value of the probe spacing was ~0.30 Å, and using this value in the calculation maximized contrast and hence SNR (see Figure S8). Scale bars, 5Å.

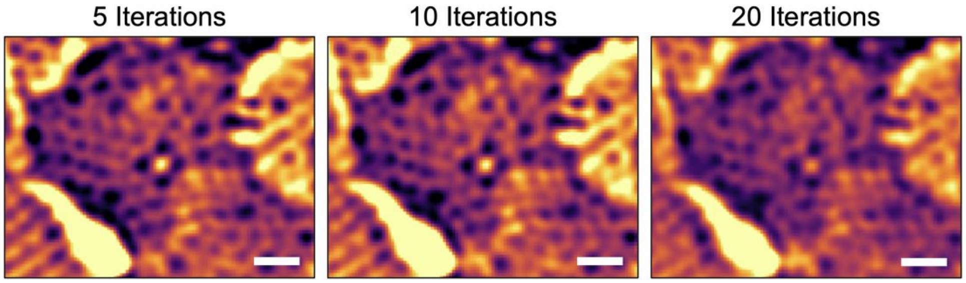

Figure S13. Comparison of 30 keV Wigner distribution deconvolution (WDD) results with varying numbers of aberration diagnosis iterations. For each, the number of iterations was applied to 1st order Bragg reflections, then 1st and 2nd order together, then 1st, 2nd, and 3rd order together. Contrast, and hence SNR (see Figure S8) was enhanced slightly up to 10 iterations and then decreased by 20 iterations. Scale bars, 5 Å.

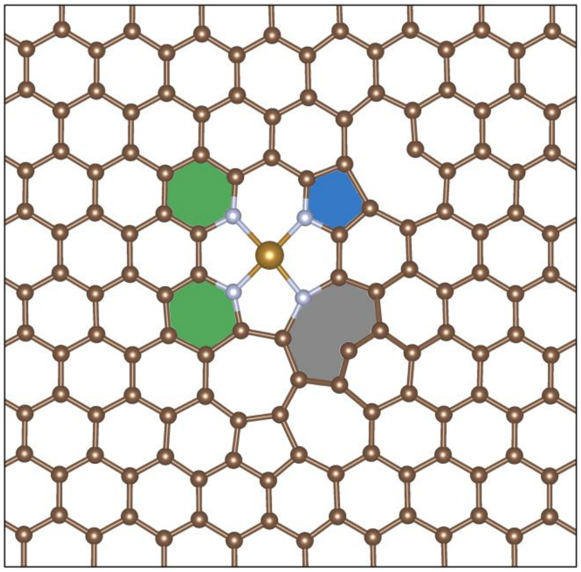

Figure S14. Stable site structure containing a C8 ring, in addition to two C6 rings and one C5 ring, calculated based on experimentally observe site. A stable structure was found that matches the experimentally observed rings adjacent to the metal atom, which dominate the electronic and oxygen reduction reaction (ORR) properties. DFT calculations revealed that this site strongly over binds ORR intermediate species, with the last protonation step determining a potential of 0.14 V vs. CHE. This structure is also a strong candidate for spontaneous ligation given its over binding nature and local defects, which may provide easier access to the axial fifth position without blocking surface reactivity. Paired with the experimental observation of a site such as this, non-ideal sites with inferior ORR characteristics are present in our well-controlled CMS material, indicating that understanding the presence and properties of sites such as this in a variety of CMS materials is critical to fully optimizing their properties.

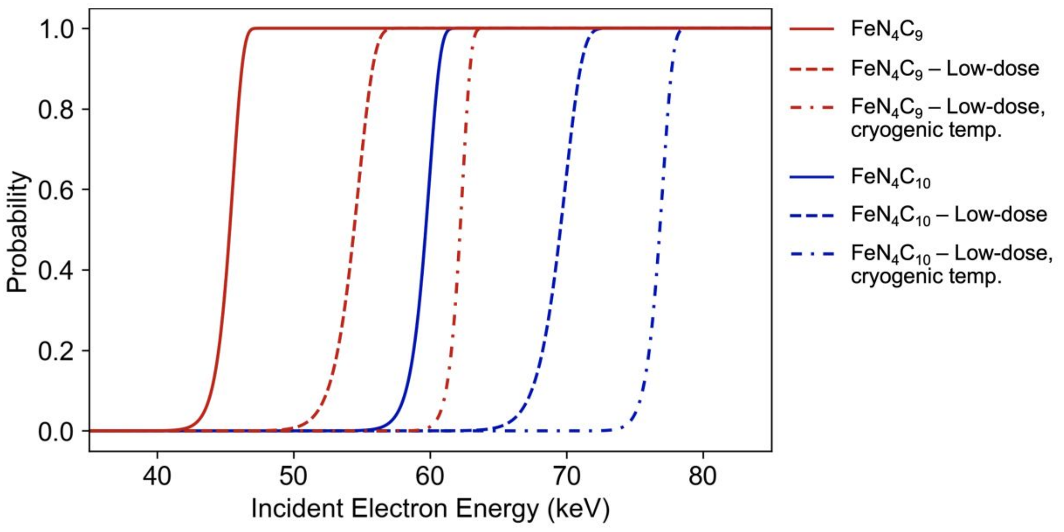

Figure S15. Effects of low-dose and cryogenic techniques on cumulative damage probabilities. Both low-dose and cryogenic techniques increase the probe energy that can be safely used. Combined, these techniques could increase the threshold probe energy by nearly 20 keV, potentially allowing standard 60 keV probes to be used, and hence resolution improved, with only a small probability of damage for the most sensitive sites. These techniques bring a host of additional challenges, however, including very low signal-to-noise ratio data and the potential for ice contamination or deposition onto the sample from the instrument vacuum. Here, we define standard dose as 10^8 $e^-/Å^2$ and low-dose conditions as 10^2 $e^-/Å^2$. Standard temperature here is defined as 300 K, while cryogenic temperature is defined as 100 K.

Parameter	Value
Beam Energy	30 keV
Probe Semi-convergence Angle	28.0 - 35.0 mrad (40 - 50 μm apertures)
Probe Current	25 - 40 pA
Probe Spacing	0.20 - 0.30 Å
Scan Dimensions	128 x 128 (real space)
Probe Dwell Time	1 ms
Camera Length	10 cm
Detector	pnCCD (no binning)
Algorithm Used	Wigner Distribution Deconvolution with Aberration Correction (PtychoSTEM MATLAB Package)
Bragg Reflections Used for Aberration Diagnosis	1st and 2nd order
Aberration Diagnosis Iterations	10 iterations: 1st order aberrations<br>10 iterations: 1st, 2nd order aberrations<br>10 iterations: 1st, 2nd, 3rd order aberrations
Number of Trotters for Aberration Diagnosis	10
Epsilon Ratio	1.0
CCD Distortion Correction Model	'30kV_Tachikawa'

Table S1. Parameters for optimized 30 keV electron ptychography reconstructions.
